# Triggers of treatment interruption and resumption among individuals with type 2 diabetes: a narrative cross-sectional qualitative study

**DOI:** 10.1080/17482631.2025.2496181

**Published:** 2025-04-29

**Authors:** Tomoo Hidaka, Rieko Suzuki, Katsue Hashimoto, Mariko Inoue, Shota Endo, Takeyasu Kakamu, Mariko Gunji, Koichi Abe, Tetsuhito Fukushima

**Affiliations:** a Department of Hygiene and Preventive Medicine, Fukushima Medical University, Fukushima, Japan; b Health Promotion Division, Koriyama City Public Health Center, Fukushima, Japan; c CMRQ Development Division, Novo Nordisk Pharma Ltd., Tokyo, Japan; d Director, Koriyama City Public Health Center, Fukushima, Japan; e Department of Orthopedic Surgery, Igarashi Clinic of Medicine and Surgery, Fukushima, Japan

**Keywords:** Treatment cessation, patient dropouts, thematic analysis, commitment, public health nurse, secondary prevention

## Abstract

**Purpose:**

Treatment interruption and resumption are common among people with type 2 diabetes mellitus (T2D), but the triggers of resumption, according to the reasons for interruption, remain underexplored. This study examined patterns of treatment interruption and resumption.

**Methods:**

Narratives from 13 T2D patients with a history of treatment interruption were analysed through semi-structured interviews.

**Results:**

Four patterns were identified: 1) “Economic rationality”, where financial barriers caused interruptions, but resumption was facilitated by low-cost check-ups and updated patient mindsets to manage medical expenses within the constraints of a limited household budget; 2) “Proactive information seeking”, where doubts about treatment effectiveness led to interruptions, followed by resumption through active health risk reassessment by the patient’s self-directed efforts; 3) “Health professional-patient relationship”, where conflicts with healthcare providers prompted interruptions, but trustful encounters encouraged resumption; and 4) “Sustained partnerships with community health professionals”, where personal challenges caused interruptions, but non-coercive partnerships with community health professionals fostered resumption through strengthened patient commitment.

**Conclusion:**

This study highlights the need for tailored medical support and local policy development for T2D patients, emphasizing subjective interpretations of their experiences on treatment interruption and resumption. Recognizing these patterns can guide resource allocation and the design of community-based diabetes care interventions.

## Introduction

There are 540 million people with diabetes worldwide, 90% of whom have type 2 diabetes mellitus (T2D) (International Diabetes Federation, 2021). T2D is a severe risk factor for reduced physical function and quality of life and contributes to increased mortality and healthcare costs (Centers for Disease Control and Prevention, 2024; Khan et al., 2020; Parker et al., 2024). The global prevalence of T2D is expected to reach 9.5% by 2050 (GBD 2021 Diabetes Collaborators, 2021).

Continuous treatment to control blood glucose levels is necessary to prevent the worsening of T2D. In other words, treatment interruption triggers a serious health crisis. Previous studies indicated that treatment interruption leads to an increase in HbA1C levels (Wu et al., 2012), consequently elevating the risk of complications such as cardiovascular disease, neuropathy, retinopathy, and nephropathy (Okemah et al., 2018), and further fatal events such as stroke and acute myocardial infarction (Rawshani et al., 2018). Moreover, treatment interruption is associated with a decline in physical function (Awotidebe et al., 2017), emotional distress due to decreased self-efficacy (Gonzalez et al., 2015), and social isolation due to negative interpersonal relationships (Rivers & Sanford, 2024). From a socio-economic perspective, interruption of T2D treatment is a risk factor for increased healthcare utilization, including rehospitalization and emergency visits (Wu et al., 2012), and decreased labour productivity, such as absenteeism (Genovese & Tedeschi, 2013).

Previous studies have identified factors that encourage people with T2D to adhere to treatment, such as rich health literacy (Al-Qerem et al., 2024), psychological acceptance of the disease (Świątoniowska-Lonc et al., 2021), trust in doctor (Pereira et al., 2020), and the presence of a future perspective that gives meaning to treatment (Hidaka et al., 2021). In contrast, factors that lead to nonadherence include financial poverty (Rezaei et al., 2019), complexity of the treatment plan (Morello & Hirsch, 2017), and distance from the healthcare facility (Graber et al., 1992).

Importantly, even patients who temporarily discontinue treatment can return to treatment. Resuming treatment after an interruption is common, and previous studies have suggested that patient perceptions, such as worsening HbA1C and risk of complications (Tominaga et al., 2021), or social relationships, such as persuasion by healthcare professionals, friends and/or family (Kalirai et al., 2020), are factors in this resumption.

However, the triggers of resumption according to the reasons of interruption remain underexplored. In other words, the combined patterns of reasons for interruption and resumption are need to be explored. By investigating these patterns, it would be possible to extract events that serve as turning points for treatment resumption based on a patient’s life history. Such an investigation will provide underlying evidence for allocating medical support resources for patients with T2D.

This study aimed to explore the combined reasons for treatment interruption and resumption among patients with T2D using qualitative methods.

## Material and methods

### Setting and sampling

The data in this cross-sectional study are consistent with those of our previous research projects (Hidaka et al., 2021, 2023). Using purposive sampling, we identified 110 potential participants aged 40 years or older living in Koriyama City, Fukushima Prefecture, Japan, as of 2018. At the time of the study, the total population of Koriyama City was 329,903 and adults aged 65 years or older accounted for 28.2% of the total population, which is similar to the national average in Japan (28.9%). Given this demographic composition, Koriyama City was considered representative. Participants were selected from each area of Koriyama City based on the population ratio. All participants had a history of T2D, as recorded in the Fukushima National Health Insurance Organization database.

Of the 110 potential participants, four were excluded because their self-reported treatment history differed greatly from the objective medical records or because it was unclear whether they had been diagnosed with T2D. Among the remaining 106 participants, 89 were treatment continuers. Of these, 13 individuals who had previously interrupted treatment but later resumed it were included in the final analysis.

It should be noted that this study used subjective reports of the presence or absence and the duration of treatment interruptions. There may have been cases in which a patient went to the hospital and was given medication but did not take it. Such cases are objectively classified as treatment continuation in the database, but are actually treatment interruptions. Therefore, only subjective reports were included in this study.

### Procedure and measurement

In October and November 2018, public health nurses from Koriyama City Health Centre conducted one-on-one interviews with the participants in the places according to the participants’ preferences. Before the interviews, the first author, who specializes in qualitative research in psychology, trained the public health nurses on conducting the interviews to refine the procedures and questions. The interviews were conducted using an interview guide prepared by the first author, under the supervision of the fifth and sixth authors, both medical doctors with specialized knowledge in diabetes.

In order to specifically confirm the interruption/resumption of treatment for each participant, the semi-structured interviews were conducted using the following six key questions based on the patient’s medical history: “When was diabetes first diagnosed?”; “Please tell me about the circumstances that led to the diagnosis”; “How did you feel when you were diagnosed with diabetes?”; “Have you ever gone for more than six months without going to the hospital or taking medication for blood sugar?”; and “Please tell us about the circumstances that led to the interruption/resumption of treatment”. Taking advantage of the characteristics of the semi-structured interview method, the interviewers posed follow-up questions to patients about their stated reasons for treatment interruption/resumption, such as, “If reason X had not been present, what do you think would have happened?” or “Was that reason the decisive factor?” This interviewing setup allowed for greater clarity in uncovering the structure of subjective reasoning and the narrative process leading to treatment resumption.

In addition, the duration of interruption, time since resumption, current type of treatment, and current blood sugar levels were estimated from the patients’ reports. As shown in the questions above, six months without hospitalization or medication was used as the criterion for treatment interruption, in line with previous studies on treatment persistence in T2D (Cramer et al., 2008; Roborel de Climens et al., 2020; Simmons & Fleming, 2000).

Each participant was interviewed once and the average length of the interviews was 29.4 minutes. With the participants’ permission, each interview was digitally recorded, transcribed verbatim by a professional, and analysed by the authors.

### Qualitative analysis

Prior to coding, we read through the verbatim transcripts and broadly identified the following four domains along a time course: “diagnosis of T2D”, “responses at diagnosis”, “reasons for interrupting treatment”, and “reasons for resuming treatment”. These domains were identified by investigating the structure of subjective reasoning and processes related to treatment interruption and resumption, which aligned with the study’s purpose. For narratives in which this structure was not immediately clear, we reviewed the interview dialogue’s preceding and subsequent segments or the entire flow. In fact, in some cases, participants did not provide a clear response to the interviewer’s questions immediately but instead recalled relevant experiences and described them in detail later in the interview. Therefore, these procedures were deemed appropriate for a careful and nuanced analysis of the narratives.

To classify the narratives according to the above-mentioned four domains, we conducted inductive coding of the interview transcripts using hybrid thematic analysis (Boyatzis, 1998), assigning labels to each transcript fragment with similar underlying meanings that could be integrated into a larger concept. In this inductive coding, the recurring terms and themes within the narratives were focused. For example, statements such as “I have no money”, “I only have my pension”, and “I’m struggling financially” were repeatedly observed across interviews. These narratives were grouped under the thematic category of economic barriers, which represented the reason for treatment interruption.

Importantly, we organized an individual’s reasons for interrupting and resuming treatment to identify their patterns, combining themes in the domains, “reasons for interrupting treatment” and “reasons for resuming treatment”. Four patterns were ultimately identified: “economic rationality”, “proactive information seeking”, “health professional-patient relationships”, and “sustained partnerships with community health professionals”.

To deepen our understanding of the patterns generated through analysis and to evaluate their appropriateness, we examined the social and cultural backgrounds and the contexts that influenced the formation of the narratives. For instance, in the “economic rationality” pattern, narratives included statements indicating difficulty affording medical expenses due to having only a pension as a source of income. The labour system in Japan strongly shapes this type of narrative. In Japan, although this may vary by occupation, mandatory retirement is typically required at the age of 60 or 65, making it difficult to continue full-time employment and resulting in pensions becoming the primary source of income (Kitamura & Adachi, 2024). Such changes in employment conditions lead to a reduction in income. Among patients with T2D who were unable to accumulate sufficient financial resources before retirement, treatment interruption due to an inability to pay for medical care tends to occur. The “economic rationality” pattern was considered valid in light of these socio-economic contexts. Similarly, the content was examined in terms of context at the narrative level for other patterns and themes.

To enhance trustworthiness, validity and reliability in a qualitative study, we employed peer debriefing for the present study. We offered an experienced qualitative researcher who was not directly involved in this research project but was affiliated with the same department as the first author to minimize the conflicts of interest; note that the involvement of outside researchers was ethically difficult in the present study. The experienced qualitative researcher reviewed and evaluated the transcripts, methodology, and findings from the viewpoints of vagueness, over/underemphasis, bias of the descriptions, and general errors and readability.

We developed an audit trail to ensure transparency in the research process, particularly the analytical process (see supplementary Table S1). We adopted the structured format proposed by Carcary (Carcary, 2020), which provides a systematic framework. In this format, the research process is documented across two dimensions, physical and intellectual, with the following subcategories included: Research Problem Identification, Research Proposal Development, Literature Review, Research Framework Definition, Sample Selection, Evidence/Raw Data Collection, Evidence Management and Analysis, and Artefact Development for physical audit trail; then, Clarification of Philosophical Stance, Consideration of Alternatives for Evidence Collection and Data Analysis, and Evidence Interpretation for intellectual audit trail.

All qualitative analyses were performed manually using Microsoft Excel (2019 MSO, version 16.0).

### Saturation

In this study, saturation was assessed by the relevant framework. We employed the concept of information power (Kauppila et al., 2016), a framework for assessing qualitative study’s sample size and saturation based on the following five dimensions: study aim (narrow/broad), sample specificity (dense/sparse), established theory (applied/not), quality of dialogue (strong/weak), and analysis strategy (case/cross-case). According to this framework, studies with a narrow aim, a dense (specific) sample, an established theory, high-quality dialogue, and a case-focused analysis strategy are considered to have greater information power and, therefore, justify smaller sample sizes compared to studies that do not meet these conditions.

For the present study, regarding the study aim, we focused on exploring the combination of reasons for treatment interruption and resumption. Compared to studies examining only one of these aspects of treatment continuity, our focus was regarded as relatively limited (narrow). Regarding sample specificity, a previous study indicated that approximately 10% of individuals with T2D experienced treatment interruption (Malterud et al., 2016), suggesting that our sample consisted of a small and specific subset of this population (dense). In terms of established theory, the present study did not adopt a top-down application of theory due to the exploratory nature of the study design (none). Concerning the quality of dialogue, the interviews were conducted by the public health nurses who had established a trusting relationship with the patients, received prior interview training, and used the interview guide. The private settings, such as the patients’ homes, were employed for the interviews. The dialogues were of high quality (strong). Finally, regarding the analysis strategy, the study aimed to explore the combinations of reasons behind treatment interruption and resumption, thereby focusing on in-depth, case-oriented analysis (case) instead of cross-case analysis. Therefore, from the perspective of information power, a small sample size was deemed appropriate in the present study.

Note that we prioritized whether the sample size and data collection were sufficient to yield meaningful insights. Given that potential study participants with a history of treatment resumption following interruption and who could articulate their illness experiences in depth were limited, this prioritization was rational. Thus, no additional recruitment was conducted.

Based on the above-mentioned theoretical assessments using the concept of information power, saturation was deemed to have been achieved. The final number of interviewees was determined to be 13, and the analytical results derived from their narratives were presented as the study’s outcomes.

### Ethical considerations

This study was approved by the Ethics Committee of Fukushima Medical University (Application No. 30196). Our study was performed in accordance with the Helsinki Declaration of 1964 and its later amendments, and all participants provided written informed consent to participate in the present study. A written informed consent to publish was also obtained from all participants.

## Results

Table I presents the participant profiles. The participants included 11 males and 2 females. The median (25th-75th percentile) age was 68 (66–70) years at the time of the interview and 51 (50–60) years at the time of T2D diagnosis, and the disease duration was 15 (10–20) years.

**Table I.** Interviewee profiles.

Interviewee	Sex	Age at interview/diagnosis (years)	Disease duration (years)	Maximum duration of treatment interruption	Timing of treatment resumption	Type of treatment	Blood sugar levels^a^
A	Male	70/50	20	17 years	3 years ago	Oral medication	100–150^b^
B	Female	59/57	2	Half a year	A year ago	Oral medication, diet, and exercise	6.0
C	Male	60/50	10	Unidentified	Several years ago	Oral medication	6.1
D	Female	70/60	10	Several years	3–4 years ago	Oral medication	6.0
E	Male	71/60	11	1.5 years	A year ago	Oral medication, diet, and exercise	7.7
F	Male	68/51	17	Several years	Several years ago	Oral medication and diet	5.8
G	Male	66/52	14	A year	A year ago	Oral medication, diet, and exercise	Normal^c^
H	Male	63/33	30	13 years	17 years ago	Oral medication and diet	6.7
I	Male	68/66	2	2 years	This year	Oral medication and diet	100–180^b^
J	Male	69/67	2	Half a year	Half a year ago	Oral medication and exercise	7.0
K	Male	68/50	18	14 years	4 years ago	Oral medication	<5.6
L	Male	71/51	20	Several years	Several years ago	Oral medication	130^b^
M	Male	68/48	20	19 years	A year ago	Oral medication and diet	6.5

Age at diagnosis, disease duration as at time of interview, maximum duration of treatment interruption, and timing of treatment restart were estimated from the interviewees’ reports.

^a^The blood sugar levels were approximate values based on patient self-reports, and unless otherwise noted, they were measured by HbA1C.

^b^The blood sugar levels were measured by fasting blood sugar.

^c^The patient forgot the exact value but reported that their blood sugar level was within the normal range.

For the results of qualitative analysis, the narratives were gradually summarized, and the numbers of initial labels and themes were 13 to 3 for “diagnosis of T2D”, 13 to 4 for “responses at diagnosis”, 13 to 4 for “reasons for interrupting treatment”, and 13 to 4 for “reasons for resuming treatment”. The detailed explanations of themes were indicated in Table II for “reasons for interrupting treatment” and “reasons for resuming treatment” and in supplementary Table S2 for “diagnosis of T2D” and “responses at diagnosis”. In Table II, the domains “reasons for interrupting treatment” and “reasons for resuming treatment” represented the subjective explanation of the kind of circumstance, condition, or background that triggered treatment interruption/resumption. The combined patterns of reasons for interrupting/resuming treatment are shown in Table III.

**Table II.** Theme by domain and explanation.

Domain	Theme	Explanation
Reasons for interrupting treatment	Economic barriers	Difficulties in paying for medical expenses due to financial reasons such as having only a pension or losing a job.
	Doubts on the effectiveness of the treatment	The lack of areal sense of the impact of taking medication or different therapies on improving T2D, or excessive belief in the benefits of unconventional (non-standard) therapies including supplements.
	Conflict with healthcare professionals	Dissonance in the relationship with the healthcare professionals, resulting from experiences such as being accused of skipping hospital visits or being distracted from treatment due to the professional’s excessively permissive nature.
	Hassles, laziness, and busy schedules	Abandoning regular hospital visits due to personal such as atendency to avoid hassles and laziness, as well as abusy work schedule.
Reasons for resuming treatment	Low-cost/free health check-up and review of healthcare cost	Rethinking the importance of T2D management and the household financial allocation of medical costs triggered by providing low-cost or free health screening opportunities that compensate for economic deprivation.
	Active opportunity to objectively assess the health crisis related to T2D	Awareness of the T2D condition at acritical level, based on the patient’s initiative to understand the significance of the objective test results.
	Health professional-patient relationships that create adesire to seek care	Encountering ahealthcare professional, such as adoctor, who encourages the patient and brings asense of security and hope.
	Respectful healthcare advice within asustained partnership	Relationships with healthcare professionals who are enthusiastic reassure the patient about the need for treatment and increase the patient’s commitment to treatment.

**Table III.** Patterns of reasons for treatment interruption and resumption with narrative examples.

Pattern	Reason for interruption	Reason for resumption	Interviewee	Narrative examples	
				For interruption	For resumption
Economic rationality	Economic barriers	Low-cost/free health check-up and review of healthcare cost	A, B	“I don’t get a lot from my pension. Financial concerns impacted my treatment. For instance, there was a period when I couldn’t go to the hospital for almost a month because I didn’t have the money.” (Interviewee A)	“I got a notice from Koriyama City (Public Health Centre) saying there was a free health check-up on offer. I thought I’d go for it as it was free. When I went for the check-up I found that my blood sugar levels were higher than before and I realized that I was in desperate need of treatment. I’m still struggling financially, but by reviewing my household finances, I’m starting to get my health under control.” (Interviewee A)
Proactive information seeking	Doubts on the effectiveness of the treatment	An active opportunity to objectively assess the health crisis related to T2D	C, D, E, F	“I stopped the treatment at the hospital because I found a supplement that’s said to be effective for diabetes. As I continued taking it, I noticed my condition improving day by day. I didn’t think I needed to go to the hospital and pay a lot of money for medicine because the supplement was so effective. I didn’t think my hospital visits and medication were worth it.” (Interviewee E)	“I had been getting regular health check-ups, so I was aware of my blood sugar levels; my HbA1c was double digits. I researched the health risks associated with my blood sugar levels, and came across an online article stating, ‘You won’t live to be 70’. I realized I was in a critical situation, especially since the article provided objective medical evidence. I made an appointment at the hospital and started going back for visits.” (Interviewee E)
Health professional-patient relationship	Conflict with healthcare professionals	Healthcare professional-patient relationships that create a desire to seek care	G, H	“There was a time when I couldn’t move properly due to lower back pain, and I couldn’t keep up with my diabetes treatment. After my back pain got better, I went to the doctor. The doctor criticized me for skipping the treatment. I felt really uncomfortable. Since then, I stopped going to the hospital.” (Interviewee G)	“I used to go to a big hospital, but I stopped due to the reason I mentioned previously. Later on, when I went to a different clinic for another illness, I also restarted my diabetes treatment. My doctor now is a really good person who encourages me. I am determined to work diligently on my treatment for diabetes.” (Interviewee G)
Sustained partnership with community health professionals	Hassles, laziness, and busy schedules	Respectful healthcare advice within a sustained partnership	I, J, K, L, M	“When I was diagnosed with T2D, I was really busy at work. I probably should have kept going to the hospital, but I just didn’t have the time. To be honest, I was just too lazy to make the effort.” (Interviewee K)	“Ms X, a public health nurse, enthusiastically encouraged me to visit the hospital again. She invited me several times. The last time I saw her, I said, ‘OK, I’ll go’. Having promised to go to my appointments, I went to the hospital and resumed my treatment.” (Interviewee K)

### The “economic rationality” pattern

The “economic rationality” pattern comprised participants who had interrupted the treatment due to economic barriers but resumed the treatment after low-cost/free health check-ups and then reviewed healthcare costs. The following narrative describes how a low-cost/free health check-up contributed to the resumption of treatment and then led to acquiring the idea of raising money for healthcare by reviewing/rearranging current household finances:

I got a notice from Koriyama City (Public Health Centre) saying there was a free health check-up on offer. I thought I’d go for it as it was free. When I went for the check-up I found that my blood sugar levels were higher than before and I realized that I was in desperate need of treatment. I’m still struggling financially, but by reviewing my household finances, I’m starting to get my health under control. (Interviewee A, 9th November 2018)

### The “proactive information seeking” pattern

The “proactive information seeking” pattern included participants who had interrupted their treatment due to doubts about its effectiveness, such as concerns about hospital visits and medication, and then resumed treatment after having the opportunity to objectively assess their health crisis related to T2D. The following narrative exemplified the patient’s story of resuming treatment after recognizing that he was at extremely high risk of T2D based on objective information on the internet:

I had been getting regular health check-ups, so I was aware of my blood sugar levels; my HbA1c was double digits. I researched the health risks associated with my blood sugar levels, and came across an online article stating, “You won’t live to be 70”. I realized I was in a critical situation, especially since the article provided objective medical evidence. I made an appointment at the hospital and started going back for visits. (Interviewee E, 31st October 2018)

### The “health professional-patient relationship” pattern

The “health professional-patient relationship” pattern comprised participants who resumed the treatment after meeting a healthcare professional who encouraged and motivated them, but who also had interrupted treatment due to conflicts with healthcare professionals; for example, a doctor accused a patient of being lazy for non-adherence, despite the patient being unable to attend the hospital due to poor health, and the patient therefore felt distrust towards the doctor. The following narrative expressed how, after experiencing conflict and mistrust with health professionals, meeting a reliable and good doctor became the reason for resuming treatment:

I used to go to a big hospital, but I stopped due to the reason I mentioned previously. Later on, when I went to a different clinic for another illness, I also restarted my diabetes treatment. My doctor now is a really good person who encourages me. I am determined to work diligently on my treatment for diabetes. (Interviewee G, 7th November 2018)

### The “sustained partnership with community health professionals” pattern

The “sustained partnership with community health professionals” pattern comprised of participants who had interrupted the treatment due to personality- and life-related reasons such as laziness and busy schedules and then resumed treatment due to the enhanced motivation for treatment arising from the sustained non-coercive partnership with community health professionals. The following narrative shows that such a partnership enhanced psychological commitment to the treatment in the participant and led to the resumption of treatment, despite the intimidating feeling of hospital visits again:

Ms X, a public health nurse, enthusiastically encouraged me to visit the hospital again. She invited me several times. The last time I saw her, I said, “OK, I’ll go”. Having promised to go to my appointments, I went to the hospital and resumed my treatment.(Interviewee K, 24th October 2018)

## Discussion

The present study aimed to explore the combination of reasons for treatment interruption and resumption among patients with T2D using qualitative methods. The following four patterns were found: in the “economic rationality” pattern, participants resumed treatment after low-cost/free health check-ups and then reviewed the household finances to secure the cost of treatment, in spite of treatment interruption due to economic barriers; in the “proactive information seeking” pattern, participants resumed treatment after having the opportunity to objectively assess their health crisis, whereas they had interrupted treatment due to doubts about the its effectiveness; in the “health professional-patient relationship” pattern, participants resumed treatment by meeting a healthcare professional who encouraged and motivated them, but they had interrupted treatment due to conflicts with healthcare professionals; in the “sustained partnership with community health professionals” pattern, participants resumed treatment due to the enhanced motivation for treatment arising from the sustained non-coercive partnership with community health professionals, while they had interrupted treatment due to personality- and life-related reasons such as laziness and busy schedules. This study provides a knowledge base for allocating medical support resources to patients with T2D in local community settings and their resources.

### Detailed discussions of patterns and differences from previous studies

The “economic rationality” pattern highlighted that economic barriers, such as medical costs, hindered hospital visits and thus led to treatment interruption. This result is consistent with a previous study on T2D treatment interruption (Rezaei et al., 2019). In contrast, our results suggest that low-cost or free health check-ups can serve as a turning point in changing the mindset of patients with T2D regarding economic barriers. As shown in interviewee A’s narrative, these health check-up opportunities may provide patients a chance to think more concretely and health-consciously about how to raise funds for treatment by reviewing and rearranging household finances, instead of avoiding going to the doctor for financial reasons. While past studies revealed that low-cost/free health check-ups contributed to the improvement of self-management skills and health status among patient with T2D (Woodward et al., 2024; Zhao et al., 2022), our results may explain in detail the psychological mechanism of how such improvements in these past studies occur. A change in mindset, such as reviewing and rearranging household finances to secure medical expenses, may be an important outcome of low-cost/free health check-ups, since such a change in mindset is likely to have a positive impact on persistent treatment. In addition to promoting low-cost/free health check-ups, providing advice on allocating household budgets for diabetes management in line with economic circumstances may further reduce the number of patients with T2D who discontinue treatment owing to financial barriers, and subsequently increase treatment resumption. This advice is expected to be implemented by various local governments as an additional measure for T2D support.

The “proactive information seeking” pattern describes that the absence of feeling of the effectiveness of standard treatment led to interruption, mediated by excessive reliance on alternative health-promoting options such as commercially available supplements. This result is consistent with past studies that revealed associations between the absence of feelings of treatment effectiveness and interruption (Nombela Manzaneque et al., 2019; Świątoniowska-Lonc et al., 2021). However, our results provide concrete insights into the types of information that contribute to the resumption of treatment; as shown in the narratives of interviewee E, objective information that helps patients to understand the outcomes of T2D, such as life prognosis, according to the individual’s health condition, may be an option. In previous studies, family members have played an important role in improving the self-management skills and treatment adherence of patients with T2D by providing social support (Busebaia et al., 2023; Mayberry & Osborn, 2012). However, it may be difficult for close relatives to address critical T2D outcomes and life prognosis because they are related to death. The internet has become a key resource for patients with T2D to obtain information on methods of blood glucose control, complications, and basic mechanisms of diabetes (Weymann et al., 2016). It may also work as a platform for patients to actively explore topics related to death, of which relatives close to the patient may find difficult to discuss. Importantly, low-quality T2D-related information is often disseminated on the internet, such as information contrary to the evidence or with little warrant (Basinger et al., 2019; Gimenez-Perez et al., 2020; Keselman et al., 2021). Therefore, public organizations such as local governments and public health centres must become quality-assured sources of information.

In the “health professional-patient relationship” pattern, treatment interruption was found to be associated with poor relationships with health professionals; for example, there was conflict and distrust with the doctor. As shown in his narrative, interviewee G became tired of being accused of non-adherence by the doctor and then interrupted his treatment. However, he resumed treatment after meeting a reliable doctor who encouraged him to continue treatment. A previous study indicated that a trusting relationship with healthcare professionals was a factor for continuous treatment engagement in T2D (Pereira et al., 2020). Our results suggest that such a trusting relationship with health professionals contributes to treatment resumption in addition to treatment continuation, as mentioned in the previous study. Communication that includes encouragement in the health professional-patient relationship is fundamentally associated with providing patients with hope and a sense of security (Edelman et al., 2019). Health professionals need to understand that encouraging patients with T2D, instead of accusing or criticizing them, may positively affect treatment resumption and subsequent continuation when interacting with patients who have interrupted treatment.

The “sustained partnership with community health professionals” pattern highlighted that the sustained partnership between community-based health professionals and patients contributes to treatment resumption, whereas personality- and life-related factors hinder treatment engagement. As shown in interviewee K’s narrative, enthusiastic but not forced advice from a public health nurse or community-based health professional to see a doctor promoted treatment resumption. Importantly, interviewee K expressed the psychological process in which he needed to go to the hospital because he promised the public health nurse that he would restart treatment. This expression suggests that a commitment, a concept of behavioural economics that maintains self-consistency through keeping a promise (Baca-Motes et al., 2013; Strecher et al., 1995), was established, leading him to resume hospital visits. A previous study indicated that the activities of community-based health professionals, such as public health nurses, benefit from improving disease-related knowledge, physical health, and self-care behaviour in patients with T2D (Trump & Mendenhall, 2017). In addition to the benefits, public health nurses also seem to play an important role in supporting patients with T2D in the community to resume treatment. Public health nurses may strengthen patients’ commitment to resuming treatment by scheduling hospital visits with them, as agreeing on a specific schedule together creates a commitment or promise. Although commitment is an internal psychological process within an individual patient, the trigger for starting such a process is an external environmental factor such as a sustained partnership with health professionals.

### Strength of the present study

Our findings provide insights into the psychological mechanisms underlying treatment resumption behaviours. While previous studies have often been limited to a superficial exploration of these behaviours, the present study explored patients’ subjective interpretations, highlighting their importance and functionality. These outcomes were achieved through qualitative methods, which enabled a descriptive elucidation of the combination patterns of reasons for treatment interruption and resumption.

Humans have a social desirability bias that causes them to answer questions in a way that others view favourably (Krumpal, 2013). Therefore, patients with T2D may avoid reporting undesirable past behaviours, such as their treatment interruption history. This bias can lead to an underestimation of the number of patients with T2D who experience treatment interruption. This underestimation may obscure the effectiveness of health policies for promoting treatment engagement among patients with T2D. For example, the low-cost/free health check-ups in the “economic rationality” pattern and good relationships in the “sustained partnership with community health professionals” pattern in our study can be considered as successful examples of secondary prevention in local health administration, focusing on reducing health problems through early detection and intervention. However, the effectiveness of such secondary prevention measures is masked by underestimating the number of patients with T2D with a history of treatment interruption; it becomes difficult to grasp the actual situation of patients with T2D who have switched from treatment interruption to resumption through such measures. We believe that the present study provides underlying evidence for allocating medical support resources for T2D and developing diabetes policies and measures in local communities through a detailed description of individuals with a history of treatment interruption, which is often underestimated. Such evidence is considered to be useful in light of the representativeness of the sample in this study; patient characteristics such as mean age and disease duration in this study are similar to those in Japan as a whole (Ohsugi et al., 2021), where the study was conducted, supporting the transferability of the findings.

## Limitations and future studies

This study has three limitations. First, because the number of interviewees was small, 13 individuals, the results may be variable in future surveys. It cannot be excluded from the possibility that there are other treatment interruption and resumption patterns in patients with T2D. Although our research findings will help to support the resumption of treatment for T2D, we acknowledge that this lack of data saturation is a weakness and should be described as a limitation. Second, the participants in this study were people with T2D who had resumed treatment after an interruption, while those who continued to stop treatment were not included. In order to identify the determinants of treatment resumption, it was necessary to use a research design that compared these two types of participants. However, our study did not use such a research design because it focused on descriptive and qualitative clarification of the status of patients who resumed treatment, for which there needs to be more evidence. The next study should use a comparative design based on the results of our study. Third, there is room for investigating the underlying mechanisms driving the patterns of treatment interruption/resumption. For example, the future study may explore what specific aspects of “economic rationality”, such as cost-benefit analysis or financial support, facilitate treatment resumption, as well as for remaining patterns “proactive information seeking”, “health professional-patient relationship”, and “sustained partnership with community health professionals”. Furthermore, exploring the impact of contextual factors, such as healthcare system characteristics, socio-economic status, or cultural background, on treatment interruption and resumption may contribute to the improvement of T2D patient’s lives.

## Conclusion

The four patterns of treatment interruption/resumption among T2D patients were identified. The “economic rationality” pattern suggests that low-cost/free health check-ups may function as a means for financially struggling patients with T2D to review their household finances and update their mindsets, enabling them to allocate funds for medical expenses. The “proactive information seeking” pattern suggests that exposure to concrete details about severe outcomes, such as shortened life expectancy, may serve as a critical opportunity to recognize the importance of treatment resumption for T2D patients who actively seek information. Since family members often find it difficult to communicate such life-threatening information directly with the patients, public institutions need to provide accurate information through channels such as the Internet. The findings of the “health professional-patient relationship” pattern indicate that such relationships contribute to treatment resumption following interruption, in addition to treatment continuation as previous studies suggested. Encouraging communication is assumed to give patients hope and reassurance, which may motivate them to resume treatment. The “sustained partnership with community health professionals” pattern highlights the role of public health nurses. Public health nurses may enhance patients’ commitment through enthusiastic but not forced supportive actions, such as helping patients schedule their hospital visits, thereby facilitating autonomous treatment resumption.

Our findings provide foundational evidence for a descriptive understanding of the psychological and behavioural backgrounds of the process from treatment interruption to resumption among T2D patients. This evidence may contribute to improving treatment adherence and the patient’s quality of life by informing healthcare professionals of adequate approaches for treatment resumption and reducing healthcare expenditures at the societal or community level by supporting the allocation of resources for such patients. These findings were made possible through qualitative research methods to explore the often underestimated treatment interruption experiences of T2D patients.

## Supplementary Material

TableS1.docx

TableS2.docx

## Data Availability

The datasets of qualitative study generated and/or analysed during the study are not publicly available as the datasets had identifiable information of the study participants.
