# Supplementary material for: Triggers of treatment interruption and resumption among individuals with type 2 diabetes: a narrative cross-sectional qualitative study
Source: Int J Qual Stud Health Well-being. 2025 Apr 29;20(1):2496181. doi: 10.1080/17482631.2025.2496181 (PMC12044906; doi:10.1080/17482631.2025.2496181)
Supplement: TableS1.docx [file ZQHW_A_2496181_SM8680.docx]

| Table S1. Audit trail |
| --- |
| **Physical Audit Trail**  *Research Problem Identification:*  Beyond epidemiological and demographic factors, behavioural and psychological factors play a significant role in treatment discontinuation and continuation among patients with type 2 diabetes (T2D). Since the treatment interruption rate reaches 10–15%, addressing this issue requires focusing on these aspects.  *Research Proposal Development:*  An industry, academia, and government collaboration project was organised to integrate financial resources, data, and research expertise. The project aimed to explore the factors influencing treatment interruption and resumption in T2D patients and implement practical improvements.  *Literature Review:*  A systematic literature review focused on the definitions and determinants of treatment interruption among T2D patients, the prevalence of T2D, health impacts of interruption, psychological distress and quality of life (QOL) decline, economic consequences, and the role of public health professionals.  *Research Framework Definition:*  A qualitative research approach was adopted to understand patients’ experiences and decision-making processes based on their subjective meaning-making. A multidisciplinary research team was organised, including a diabetes specialist from a pharmaceutical company, a public health nurse from a local government, a preventive medicine specialist from a research institute, and a psychology researcher with 15 years of experience in qualitative studies. Semi-structured interviews were conducted with the cooperation of local public health nurses. The framework also included the development of practical recommendations for improving treatment adherence, which were fed back to the local government.  *Sample Selection:*  Participants were T2D patients residing in the community who had experienced treatment interruption and had established a trust-based relationship with public health nurses. Purposeful sampling was employed to gather suitable participants with rich information on the project theme. Since the patients meeting these criteria were limited, the sampling was carried out with a narrow focus.  *Evidence/Raw Data Collection:*  Semi-structured interviews were conducted using an interview guide developed by the research team and peer-checked to ensure quality. Public health nurses visited participants’ homes and conducted in-person interviews to elicit experiences of treatment discontinuation and resumption.  *Evidence Management and Analysis:*  Due to the small sample size (13 participants), patients’ responses were organised using Excel. Analyses linked the interview questions to participants’ responses. A hybrid thematic analysis approach was employed, involving step-by-step coding of empirical data and a subsequent review and selection of theories and concepts to interpret the coded results.  *Artefact Development:*  The research outcomes included identifying and discussing similarities and differences between the themes generated in this study and those found in previous research. Key factors facilitating treatment resumption among community-dwelling T2D patients were explored. A theoretical explanation was provided regarding how social desirability bias may affect research participation. Based on these findings, both an academic paper and a feedback report for the industry-government-academia collaborative project were produced.  **Intellectual Audit Trail**  *Clarification of Philosophical Stance:*  A narrative approach was employed to capture the participants’ subjective meaning-making.  *Consideration of Alternatives for Evidence Collection and Data Analysis:*  Analytical thinking and decision-making during the design of the research framework were documented through research memos and email exchanges among project members. The transferability of inductively derived findings from a small sample size was rationalised. Additionally, the advantages of focusing on patients who resumed treatment after interruption, rather than comparing them with those who remained non-adherent, were justified.  *Evidence Interpretation:*  Multiple peers repeatedly validated the data interpretations. Documentation was maintained regarding the analytical thinking, decisions made during the analysis, interpretations of the themes generated, and reflections on the findings. Particular attention was given to assessing the practical implications derived from the results. |
