# Supplementary material for: Triggers of treatment interruption and resumption among individuals with type 2 diabetes: a narrative cross-sectional qualitative study
Source: Int J Qual Stud Health Well-being. 2025 Apr 29;20(1):2496181. doi: 10.1080/17482631.2025.2496181 (PMC12044906; doi:10.1080/17482631.2025.2496181)
Supplement: TableS2.docx [file ZQHW_A_2496181_SM8679.docx]

Table S1. Theme by domain and explanation for ‘Diagnosis of T2D’ and ‘Responses at diagnosis’

| Domain | Theme | Explanation |
| --- | --- | --- |
| Diagnosis of T2D  Responses at diagnosis | Health check-ups at workplace/community  A part of treatment for other illness  Blood donation  Acceptance without seriousness  Within the scope of the assumption  Sense of urgency for health management  Shocked | Diagnosis in a hospital/clinic or community, followed by a health check at work where participants identify their high blood glucose and the need for detailed examination; public health centres often organise health check-ups in the community.  Diagnosis in hospital after the discovery of T2D as a result of a blood test for treating other health problems conditions such as kidney disease.  Diagnosis in hospital/clinic based on a report of a blood donation result suggesting the possibility of high blood glucose and the need for detailed investigation.  Receiving a diagnosis of T2D without being shocked, but also without a sense of seriousness.  Calm acceptance of the diagnosis, including the risks of complications, based on the genetic risk perception of T2D due to the presence of relatives with T2D.  Feeling the necessity to improve lifestyles associated with T2D, such as obesity and overeating, to prevent the progress of T2D by self-management of health.  Surprise and shock toward the diagnosis that contradicts previous perceptions, such as “I will never get diabetes” or “I am a healthy person”. |
